# Honey authenticity: the opacity of analytical reports - part 1 defining the problem

**DOI:** 10.1038/s41538-022-00126-6

**Published:** 2022-02-08

**Authors:** M. J. Walker, S. Cowen, K. Gray, P. Hancock, D. T. Burns

**Affiliations:** 1 Laboratory of the Government Chemist, Queens Road, Teddington, TW11 0LY UK; 2 Institute for Global Food Security, The Queen’s University of Belfast, Belfast, BT9 5AG Belfast, UK

**Keywords:** Business and industry, Analytical chemistry

## Abstract

The composition of honey, a complex natural product, challenges analytical methods attempting to determine its authenticity particularly in the face of sophisticated adulteration. Of the advanced analytical techniques available, only isotope ratio mass spectrometry (IRMS) is generally accepted for its reproducibility and ability to detect certain added sugars, with nuclear magnetic resonance (NMR) and high-resolution mass spectrometry (HRMS) being subject to stakeholder differences of opinion. Herein, recent reviews of honey adulteration and the techniques to detect it are summarised in the light of which analytical reports are examined that underpinned a media article in late 2020 alleging foreign sugars in UK retailers’ own brand honeys. The requirement for multiple analytical techniques leads to complex reports from which it is difficult to draw an overarching and unequivocal authenticity opinion. Thus arose two questions. (1) Is it acceptable to report an adverse interpretation without exhibiting all the supporting data? (2) How may a valid overarching authenticity opinion be derived from a large partially conflicting dataset?

## Introduction

In November 2020, the Government Chemist, the UK statutory technical appellate function for food control^1^, was asked to provide an independent secondary expert opinion on the dataset of analytical results underpinning a UK media article. The story carried the headline “*Supermarket brands of honey are ‘bulked out with cheap sugar syrups made from rice and corn’*”^2^; similar media stories recur from time to time, e.g^3–8^. The dataset stemmed from the analyses of 13 own-brand honey samples of major UK retailers, commissioned by a South American bee-keeping organisation. The UK Foods Standards Agency, FSA, supplied three certificates of analysis (CoA), representative of the dataset^9^. Herein is presented the Government Chemist’s opinion.

A European Directive (‘EU Directive’)^10^ defines honey as ‘the natural sweet substance produced by *Apis mellifera* bees from the nectar of plants or from secretions of living parts of plants or excretions of plant-sucking insects on the living parts of plants, which the bees collect, transform by combining with specific substances of their own, deposit, dehydrate, store and leave in honeycombs to ripen and mature’. The Codex Alimentarius definition^11^ is similar, substituting ‘honey bees’ for the specific species as, worldwide honey may be collected from other honeybee species. The EU Directive was implemented in each of the then member states^12^. UK Ministerial policy responsibilities on honey are with the UK Department for Environment, Food & Rural Affairs^13,14^, while general food law enforcement policy is with the FSA^15^.

Nectar is composed primarily of water, sugars, such as fructose, glucose, and other oligo- and polysaccharides, and minor constituents, such as pollen, proteins, amino acids, aliphatic acid salts, lipids, and flavouring components. Bees process the collected material with enzymes, including diastase (amylase) and invertase (α-glucosidase). Thus, honey is primarily a concentrated aqueous solution of ‘invert’ sugar (the monosaccharides glucose and fructose)^16^ and typically contains a wide range of saccharides, amino acids, proteins, organic acids, vitamins, minerals, enzymes, polyphenols and pollen. Some of these arise from honey maturation, others from the bees and some from the plants^17^. Honey composition depends on many factors including the botanical source, geographical origin, species of bee, year and season^18^. Codex and the EU Directive set certain compositional criteria. The EU Directive differentiates blossom honey (nectar honey in Codex) and honeydew honey, the latter from plant and insect secretions. Honeydew honey is also a concentrated aqueous solution of ‘invert’ sugar, albeit lower in fructose and glucose and typically darker than nectar honey; its chemical characteristics, such as pH, acidity, electric conductivity and other minor components including oligosaccharides are typically higher than in nectar honey^19^. Codex, the EU Directive, and national law stipulate various labelling options and requirements for honey in addition to general food labelling requirements to protect its authenticity^20^.

## Methods

Data from the three CoA were grouped into (a) well-established traditional techniques, (b) well-established recent techniques (e.g. some forms of IRMS), and (c) other more recent techniques. CoA data were assessed in 5 categories: (1) those where a legislative limit applies, (2) non-legislative but generally agreed limits, (3) quality defect data, (4) authenticity data and (5) other general data.

Recent primary literature and review papers were identified by literature search (Google® Scholar, Scifinder, 28.01.2020, search terms honey, authenticity, review, and more specific terms as appropriate). All data generated or analysed during this study are included in this published article (and/or its supplementary information files).

### Adulteration of honey and its detection

Anklam (1998)^17^ reviewed honey authenticity methods finding no single parameter provided unequivocal information about botanical or geographical origins. Some potentially suitable methods were identified indicating a botanical origin from flavonoids, pollen, aroma and marker compounds, although deliberate addition of readily-available known markers and the loss of volatile markers on storage may vitiate detection. It was suggested profiles of oligosaccharides, amino acids and trace elements could be used to verify the claimed geographical origin. A combination of methods with statistical data evaluation was a promising approach. Anklam also noted carbon stable isotope ratio analysis can detect honey adulterated with *C*_*4*_ sugars such as corn syrups or cane sugar (LoD 7%), particularly using the carbon isotope ratio of the honey protein fraction as an internal standard, but the addition of *C*_*3*_ sugars such as beet could not be proved since nectar generally arises from *C*_*3*_ plants. Of the 131 studies reviewed by Anklam, honey sample numbers tended to be small, generally below 30, with several up to 50 and only three between 90 and 100.

### Types of adulteration

**Fig. 1:**
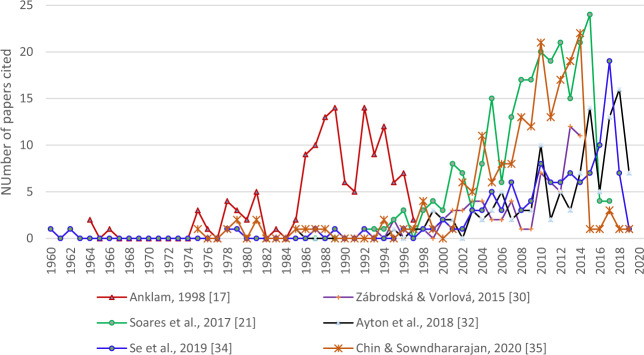
Number of papers cited by year by review articles. Coverage of original papers by review papers considered herein, *y*-axis shows numbers of papers cited; clearly the reviews by Anklam,^17^ Soares et al.^21^ and Chin & Sowndhararajan^35^ achieved more coverage that others.

**Fig. 2:**
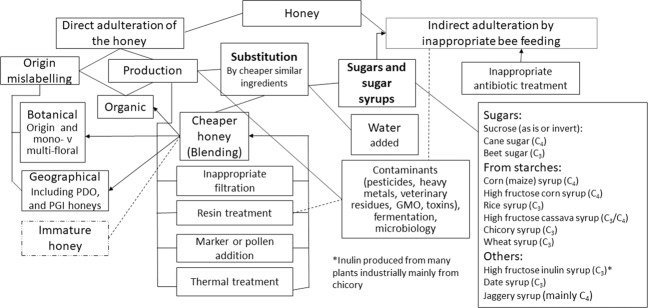
Types of honey adulteration [Sources: Anklam 1998,^17^ Soares et al., 2017,^21^ European Commission 2018,^22^ Ayton et al., 2019,^32^ Se et al., 2019^34^ and Chin & Sowndhararajan, 2020^35^].

After Anklam^17^ subsequent reviews, with variable coverage of the literature (Fig. 1) have expanded on types of adulteration (Fig. 2). The decline of bee populations has also been mentioned^21^ as a driver.

A European Commission expert stakeholder seminar of January 2018 confirmed ‘direct’ adulteration (addition of sugar/syrup) as the most frequent type of fraud. ‘Indirect’ adulteration is a term for deliberate inappropriate bee feeding with sugars when nectar is naturally available. Bee feeding is widespread and accepted when it is necessary in the absence of nectar and the expert stakeholder seminar recognised that if it does not stop when nectar becomes available it is more likely to be a malpractice rather than fraud. Adding pollen or other natural honey constituents, such as enzymes, to ultrafiltered honey and labelling it as a monofloral honey or the dilution of good quality honey with ultrafiltered honey were discussed. Synthetic resins are illegally used to remove unwanted substances (including antibiotics or pesticides) from honey, a potential health issue. Early removal of honey from the hive (immature honey) was also discussed and the Round Table report concluded “it was generally agreed that immature honey is not properly defined in legislation, and a guidance document is needed”^22^.

Views on immature honey particularly originating from certain parts of Asia are polarised. Many view systematic harvesting of immature honey followed by industrial moisture reduction as not complying with the Codex definition, since the honey is not matured by bees in the hive.^23–25^ Others point to the nomadic lifestyle of Chinese beekeepers^26–28^ and the high humidity of Asia necessitating periodic collection of immature honey for aggregation and moisture removal, to prevent fermentation. There are ongoing discussions on these issues^29^. Figure 2 illustrates the complexity of honey adulteration.

### Analytical techniques for determining honey authenticity

Methods for the detection of honey adulteration have developed in a variety of ways. Zábrodská and Vorlová (2015)^30^ considered inappropriate bee feeding difficult to detect, noting few successful studies e.g. using high-performance anion-exchange chromatography with pulsed amperometric detection, HPAEC-PAD with chemometrics, carbon isotope ratio mass spectrometry, IRMS, and gas chromatography-mass spectrometry, GC-MS. The latter identified markers such as fructosyl-fructose, although this marker has also been detected in honey from free-flying bees. One-dimensional and two-dimensional nuclear magnetic resonance spectroscopy, NMR, with multivariate analysis were regarded as effective (95.2% and 90.5% accuracy) in detecting bee feeding when applied to 63 samples of honey from various botanical sources and seven different sugar syrups marketed as specific bee-keeping products. Reviewing direct adulteration from a Czech perspective these authors^30^ noted traditional analyses^31^ and pollen analysis by microscopy (melissopalynology) are routinely applied. The latter is relatively time-consuming, although microscopy for the presence of starch grains may rapidly reveal crude addition of starch-derived syrups. Physicochemical investigations include analysis of phenolic and volatile compounds, protein, free amino acid content, colour, lactones, water activity, free fatty acids, sensory characteristics and antioxidant activity. Low honey prices in some countries with year-round production, very large broods and low labour costs are associated with honey quality indicators considerably different from those of traditional Czech honey but this may not necessarily mean lower quality. Fermentation which may occur when honey is harvested prematurely, may be obvious from the appearance or revealed by physicochemical and microbiological analyses. Pollen DNA by Polymerase Chain Reaction, PCR for botanical and geographical origins involves time-consuming and laborious DNA extraction, but one successful study was reported^30^.

Soares et al. (2017)^21^ confirmed stable carbon IRMS as the most appropriate approach to detect *C*_*4*_ sugar adulteration in honey and reviewed detection of *C*_*3*_ sugar syrups noting chromatographic approaches for oligosaccharides and polysaccharide fingerprints and advances in spectroscopic techniques. Multi-elemental and trace analysis appeared to be the most promising approach to discriminate the geographical origin of honey. Again the need for use of at least two complementary techniques and large datasets from authentic samples was noted. These authors summarised Protected Designation of Origin (PDO) and Protected Geographical Indication (PGI) honeys registered with the European Commission as of 2017 and characteristic volatile compounds are described (2012 -2015) for different types of honeys. The presence of formic, oxalic and lactic acids in honey could be attributed to their use against the *Varroa* parasite as alternatives to synthetic acaricides. The effectiveness of nontargeted NMR, Raman spectroscopy and Infrared IR spectroscopy in combination with chemometrics was regarded as having been demonstrated, but more efforts were called for to validate and include them as official methods for honey authentication. Advances were reviewed in DNA analysis, including next-generation sequencing applied to botanical and entomological authentication of honey although it is inapplicable to filtered honeys^21^.

Negative media coverage in 2018 on the alleged presence of adulterated honey in Australian supermarkets prompted an Australian government review of the Australian honey industry^32^. The review discussed the strengths and weaknesses of analytical techniques with particular focus on elemental analysis IRMS, EA-IRMS, including the causes of unusual isotopic fractionation in *Leptospermum* honeys, NMR and other spectroscopic techniques (mid- and near-infrared (MIR, NIR) and Raman). The databases associated with NMR and other non-targeted spectroscopic approaches were assessed in some detail with concerns that, when used for Australian honey samples, “typical” ranges had been established with honeys from other countries, mainly in Europe and Asia not necessarily appropriate for Australian honey. The review made recommendations aimed at regaining consumer confidence in Australia honey. More recently, improvements in the protein precipitation procedure have been reported to eliminate the apparent failure of Australian honeys in EA-IRMS testing for *C*_*4*_ sugars^33^.

Se et al. (2019)^34^ reviewed honey adulterants and the advantages and disadvantages of 17 techniques including NMR and other spectroscopies, sensor-based methods, chromatography, and marker compounds. The EA-IRMS approach of AOAC Official Method 991.41 was reviewed noting that additional HPLC-IRMS and mean Δδ^13^C HPLC-IRMS data for individual sugars successfully detected *C*_*3*_ sugar adulterants to a low level. Spectroscopic techniques with chemometrics are considerably more practical but also require correlation with traditional analytical methods and these authors recommend monitoring of honey quality via biosensor technology for the future^34^.

Chin and Sowndhararajan (2020)^35^ gave a useful summary of authentication techniques and reported the number of samples and/or honey types analysed per technique by each study reviewed. Inspection of these data (graphed for ease of reference in Supplementary Fig. 1) confirms low numbers of samples in peer-reviewed published studies persists, other than perhaps in non-NMR spectroscopic studies. By contrast commercial NMR databases contain data on over 20,000 samples^32^.

A 2015/16 European-wide honey control exercise organised by the European Commission found a substantial proportion (about 20%) of the 2264 samples taken were non-compliant owing to indications by EA-LC-IRMS of foreign sugars. However of these a much lower proportion (about 5%) of the samples taken in the UK were non-compliant, owing to incorrect botanical source (4%) or presence of exogenous sugars (1%)^36^.

The above review papers and the report of the JRC ‘Round Table’ (European Commission 2018),^22^ confirm analytical techniques to authenticate honey include the following.

1. Conventional physicochemical analysis, most of which is official and harmonised, and pollen analysis by microscopy.
2. Isotopic techniques, EA-IRMS and LC-IRMS.
3. Separation techniques, e.g. sugar profiling by LC or GC.
4. Spectrometric techniques, including LC-followed by high-resolution mass spectrometry (LC-HRMS), LC-MS/MS for marker detection and GC-MS for aroma profiling.
5. Spectroscopic techniques, including Fourier transform infrared (FTIR), NIR and NMR.
6. Trace elements profiling by inductively coupled plasma-mass spectrometry (ICP-MS).
7. Molecular biology, DNA barcoding and Next Generation Sequencing.
8. Statistical tools.
9. Other techniques such as the use of biosensors, electronic tongues and noses, and sensory analysis.

The UK Government Chemist convened a seminar on honey authenticity on 13 November 2019 on the determination of exogenous sugars by NMR. Fifty-seven stakeholders from across the UK honey supply chain, regulators (FSA, the Department for Environment, Food and Rural Affairs, Defra, and local authority enforcement), analytical service providers and expert scientific researchers attended. Whilst there was support for NMR as a diagnostic analytical tool, it was regarded by some as not yet suitable for the detection of exogenous sugars in honey for enforcement purposes, owing to lack of information on the databases underpinning interpretation of the method outputs. Others felt there was insufficient information on the results of inter-laboratory method comparisons and the scope of laboratory accreditation^37^. Suggestions made included continuing dialogue, training and guidance on the production and analysis of honey, and standardisation of the application and interpretation of NMR data for exogenous sugars in honey.

### Assessment of supplied CoA data

As recommended in a number of review articles, complementary analytical approaches representing a range of analytical techniques were exhibited within the CoA provided. The methods were badged as ISO/IEC 17025 accredited, except for NMR which appeared to have been sub-contracted and for which no accreditation status was provided. Tables 1–4 summarise the data received, the opinions of the reporting laboratory and the present authors’ comments.

**Table 1:** Data for which a legislative (L) or generally agreed (GA) limit applies to the samples in question.

Parameter	L or GA	Limit	Results			Reporting Laboratory’s Interpretation^a^	Authors’ comments
			1	2	3		
Moisture g/100 g	**L**	≤ 20	18.6	18.6	18.0	All compliant	All compliant
Sum of fructose and glucose g/100 g	**L**	≥ 60	79.0	78.6	79.5	No opinion other than the given data were detected	All compliant
Sucrose g/100 g	**L**	≤ 5	<0.5	<0.5	<0.5	No opinion other than the given datum was detected	All compliant
Diastase, DN	**L**	≥ 8	4.4	6.0	6.0	**Each noncompliant**	**Each** ***apparently*** **noncompliant**
HMF mg/kg (LC)	**L**	≤ 40	25.6	37.2	15.1	All compliant	All compliant
HMF mg/kg (NMR)	**L**		37.0	51.0	21.0	1. Compliant, 2. **Noncompliant**, 3. compliant	1. Compliant, 2. **Noncompliant**, but note discordant results. 3. Compliant
Fructose (F) g/100 g	**GA**	30 – 44	40.2	39.7	40.5	No opinion other than the given data were detected	Within acceptable range
Glucose (G) g/100 g	**GA**	22 – 40	38.8	38.9	39.0	No opinion other than the given data were detected	Within acceptable range
F:G ratio	**GA**	1 – 1.2	1.04	1.02	1.04	No opinion other than the given data were detected	Within acceptable range
Proline mg/kg	**GA**	≥ 180	190	<150	346	1. & 2. Proline not typical, adulteration might be possible 3. No opinion given other than the given datum was detected	1. & 3. are compliant; 2 is suspicious, falling below a proposed minimum of 180 mg/kg (Bogdanov & Martin 2002^38^)

Text in bold highlights results that may imply the honey was noncompliant with agreed limits.

^a^The laboratory’s opinion has been paraphrased for brevity and anonymity.

**Table 2:** Data relating to a possible quality defect.

Parameter	Results			Reporting Laboratory’s Interpretation^a^	Authors’ comments
	1	2	3		
Glycerol mg/kg	379	524	440	**Noncompliant** owing to a fermentation or a stopped fermentation	Honey glycerol found mean 137.6 mg/kg, range 50.0 - 366.2 mg/kg (Huidobro et al., 1993^39^). An upper limit of 300 mg/kg has been suggested (Bogdanov & Martin 2002^38^). The results found are higher than literature data and suggest incipient fermentation at some stage, the sensory properties should be assessed for off-tastes. Immature harvesting might be a possible explanation. Note however there is no support from the ethanol or 2,3-butandiol data nor has microbiology or microscopy for yeast cells been reported
Ethanol mg/kg	87	82	52	No opinion other than the given data were detected	Data within known ranges, Huidobro et al., 1994^40^ and 2001,^41^ A limit of 150 mg/kg suggested for Spanish & Italian blossom honey (Bogdanov & Martin 2002^38^).
2,3-butanediol mg/kg	21	<20	<20	No opinion other than the given data were detected	2,3-butanediol is a fermentation by product. These data are not significant

Bold text highlights a result that may imply the honey was non-compliant with quality standards.

^a^The laboratory’s opinion has been paraphrased for brevity and anonymity.

**Table 3:** Data relating to authenticity.

Parameter	Results			Reporting Laboratory’s Interpretation^a^	Authors’ comments
	1	2	3		
AOAC 998.12 ^13^C IRMS	-ve	-ve	-ve	Compliant no evidence of adulteration with… *C*_*4*_ sugars [^13^C δ values reported]	We concur with the reporting laboratory’s opinion. The method has a LoD of 7% and does not detect *C*_*3*_ sugars
HRMS Screening	+ve	+ve	+ve	**Suspicious** owing to the presence of some syrup markers which are untypical for honey	There was no disclosure of the identity of the markers and the reporting laboratory’s opinion appears tentative
NMR foreign sugars Univariate verificationMultivariate verification	+ve IM IM	+ve IM OM	+ve IM OM	**Non-compliant** owing to the presence of foreign sugars (IM = in model) (OM = out of model)	There was no disclosure of the identity of the sugars; 10 individual sugars, including mannose, a putative marker for syrups (Shievano et al. 2020^45^), and oligosaccharides were reported (see Tables 1 and 4) without adverse comment
Honey foreign alpha-amylase	+ve	+ve	+ve	**Non-compliant** owing to evidence of an enzyme used in the production of invert sugar syrup	Unspecified concentration, no LoQ given, somewhat equivocal opinion, peer-reviewed literature sparse
Caramel E150c/d	+ve	+ve	+ve	**Non-compliant** owing to evidence of caramel that possibly arose from its presence in a sugar syrup	No quantitative data or LoQ reported. Caramel may be added to mimic dark forest honey, a result > LoQ (5 mg/kg) considered non-compliant (Zábrodská and Vorlová 2015;^30^ also perhaps heating of starch derived syrups.
Psicose g/100 g (LoQ 0.05 g/100 g)	0.05	0.06	0.29	**Suspicious** owing to the presence of this sugar that possibly arose from the presence of sugar syrup although in rare cases, psicose is naturally occurring up to 0.3 g/100 g	Note possibility of natural occurrence and reported data for 1 & 2 are on or close to the LoQ. For 3 the result is just below a CoA cited upper limit of 0.3 g/100 g. Otherwise the literature on psicose, which is an epimer of D-fructose not found in honey (Doner et al. 1979^46^) is sparse.

Text in bold highlights results that may imply the honey was noncompliant with authenticity criteria.

^a^The laboratory’s opinion has been paraphrased for brevity and anonymity.

**Table 4:** Other general data.

Parameter	Results			Reporting Laboratory’s Interpretation*	Authors’ comments
	1	2	3		
Foreign oligosaccharides	-ve	-ve	-ve	No commentary	See note 1
β-fructofuranosidase	-ve	-ve	-ve	No evidence of adulteration with invert syrup produced with these enzymes	Agree and welcome the explanation
Gamma-amylase	-ve	-ve	-ve		
Beta-amylase	-ve	-ve	-ve		
Thermostable amylases DN	<0.1	<0.1	<0.1		
Gentibiose g/100g	<0.3	<0.3	<0.3	No opinion other than the given data were detected (applies to all data from ‘Gentibiose’ to ‘Succinic acid’)	Known to be present in honey (applies to all parameters from ‘Gentibiose’ to Succinic acid’)
Maltose g/100g	1.1	1.2	1.0		
Maltotriose g/100g	<1.0	<1.0	<1.0		
Mannose g/100g	0.0	0.1	0.1		
Melezitose g/100g	<1.0	<1.0	<1.0		
Raffinose g/100g	<0.1	0.1	<0.1		
Turanose g/100g	0.2	0.3	0.3		
Citric acid mg/kg	191	159	238		
Malic acid mg/kg	<50	<50	<50		
Quinic acid mg/kg	<300	<300	<300		
Alanine mg/kg	68	24	16		
Aspartic acid mg/kg	<150	<150	<150		
Glutamine mg/kg	<200	<200	<200		
Leucine mg/kg	44	<40	<40		
Phenylalanine mg/kg	<100	<100	<100		
Tyrosine mg/kg	<50	<50	<50		
Valine mg/kg	<10	<10	<10		
3-phenyllacetic acid mg/kg	<300	<300	<300		See note 2
Methylglyoxal, MGO, mg/kg	<30	<30	<30		
Dihydroxyacetone mg/kg	<20	<20	40		Precursor of MGO
Kynurenic acid mg/kg	<60	<60	<60		Known to be present in honey
Shikimic acid mg/kg	<80	<80	<80		
Acetic acid mg/kg	24	29	47		
Acetoin mg/kg	<20	<20	<20		
Formic acid mg/kg	21	22	52		
Fumaric acid mg/kg	<5	<5	<5		
Lactic acid mg/kg	86	41	111		
Pyruvic acid mg/kg	18	14	16		
Succinic acid mg/kg	16	18	18		

*The laboratory’s opinion has been paraphrased for brevity and anonymity.

Note 1 – oligosaccharides - Honey contains about 25 oligosaccharides (disaccharides, trisaccharides, tetrasaccharides). The presence of fingerprint oligosaccharides with a high degree of polymerisation and not found in honey can be used to detect certain syrups,^21,30^ including High Fructose Corn Syrup but not Rice syrup^17,34^.

Note 2: Marker for honey that is derived from *Leptospermum* species found in Australia and New Zealand.

A summary page in each CoA provided overall opinions for 7 categories of results for each sample, 4 containing single parameters or a single technique and 3 containing two. For all but one of the 21 categories across the three CoA, the overall opinion was “noncompliant”, including for paired results one of which was “noncompliant” and the other “compliant”. In one instance a “compliant” opinion was given, although it was not possible to see why as the individual data did not differ appreciably from those in the other two CoA.

Table 1 exhibits the data for physicochemical parameters including major sugars for which legislative or commonly agreed limits exist. Each of the three samples, two described as ‘clear Honey’ and one as ‘set honey’ were apparently deficient in diastase activity. The provisions of the EU Directive require a product described as honey to exhibit a diastase activity of not less than 8 determined after processing and blending. There is a derogation for honey with a low natural enzyme content (e.g. citrus honey) to avail of which the hydroxymethylfurfural (HMF) content must be not more than 15 mg/kg. The samples were not described as citrus honey and contained more than 15 mg/kg HMF hence the diastase numbers for each were apparently deficient of the minimum required (However, time and storage conditions could have affected the results, see further commentary in Part 2 of this series (Honey authenticity: the opacity of analytical reports—part 2, forensic evaluative reporting as a potential solution). For one sample conflicting LC and NMR results were exhibited. Data for the amino acid proline were reported and while there are no legislative limits, a minimum threshold of 180 mg/kg proline has been proposed below which dilution with exogenous sugars might be suspected^38^. On that basis, samples 1 and 3 are compliant while sample 2 at ‘<150 mg/kg’ (i.e. less than the limit of detection of the method) is not. The reporting laboratory are tentative in their opinion flagging adulteration as “possible” for both samples 1 and 2 without citing a suggested limit and despite sample 1 containing more than 180 mg/kg proline.

Table 2 exhibits data related to fermentation based on glycerol concentrations. The results found are higher than literature data^39^ and suggest incipient fermentation, for which harvesting of immature honey might be an explanation. Note however there is no supporting evidence from the ethanol^40,41^ or 2,3-butandiol data, both of which arise from fermentation. Equally, no sensory properties (off-tastes), microbiology or microscopy for yeast cells have been reported.

Table 3 exhibits data relating to authenticity, some of which appear to show non-compliance. AOAC 998.12 (^13^C IRMS) proved negative for *C*_*4*_ sugars (cane sugar or corn syrups); the method has a LoD of 7% as *C*_*4*_ sugars^42^ and does not detect *C*_*3*_ sugars. EA-LC-IRMS, capable of detecting *C*_*3*_ sugar adulteration^34,43,44^ was not carried out. HRMS screening was reported as positive for certain syrup markers which are untypical for honey. There was no disclosure of the identity of the markers and the reporting laboratory’s opinion appears tentative. NMR results were reported as positive for foreign sugars, but there was no disclosure of the identity of the foreign sugars. Ten individual sugars including mannose, a putative marker for syrups^45^ and oligosaccharides, were reported without adverse comment. Caramel was reported as positive, a non-compliance possibly indicating the presence of added sugar syrup in all three CoA but no quantitative data were given. Psicose, an epimer of D-fructose rarely found in honey^46^ was quantified in all three CoA.

Table 4 exhibits 35 data for each sample that did not excite any comment by the reporting laboratory and indeed are largely unremarkable.

On the face of it, to anyone lacking in-depth experience of honey analysis, the data in the CoA demonstrate non-compliance (for example apparent deficiency in diastase) or cast suspicion on the authenticity of the honey samples examined. This set of partially conflicting data reflects the tentative, and at times disputed, nature of much of the published work on honey authenticity, including reservations about the validity of databases. Moreover key data such as quantitative results, LoD and LoQ are in some instances missing and there is no reference to measurement uncertainty.

‘Untypical’ NMR profiles have reportedly been found in honeys derived from supply chains robustly audited as to their authenticity^32,37^ giving rise to reservations that the NMR profiles in the databases do not take account of the full range of global honeys, nor adequately reflect other variables such as seasonality. To date, although UK industry-led work is in progress (see below), there is little published evidence examining, in the above context, the adequacy or otherwise of the databases. Divergent analytical results from HRMS, IRMS and NMR on the same samples have recently been described^47^. In a small (bee keeper-led) study 14 honey samples were sent to two different analytical service providers (‘ASP1’ and ‘ASP2’). By LC-HRMS, both ASP assessed 4 samples as possibly adulterated with sugar syrup. For a further 4 samples, LC-HRMS results differed between the ASP. EA-IRMS (presumed to be AOAC 991.41) in ASP1 failed to support the HRMS results for 4 samples, and NMR in ASP2 failed to support the HRMS results for 3 samples. No sample was returned as adulterated by all three techniques in both ASP^47^.

## Conclusions

Challenges clearly remain for honey authentication and work is in progress to address these. Transparent validation of analytical methods for the determination of honey authentication, using quality assurance tools such as reference materials and proficiency testing schemes, are in development, although discrimination between industrially-dried immature honey and mature honey remains a difficult problem.

The JRC ‘Round Table’^22^ identified a need for internationally-agreed modern purity criteria for honey beyond the basic quality requirements of the current Codex and EU legislation. The ‘Round Table’ suggested a series of actions involving coordinated work from all stakeholders, including at an international level, the latter usually a lengthy process.

Progress has been made on the recommendations arising from the UK honey NMR seminar^37^, including training materials (which remain in development at the time of writing) and other aspects identified in a recent FSA publication^15^. Industry-led research is underway, including construction of a database of NMR spectra from samples relevant to the UK market. The work is said to include an investigation of NMR signals that with chemometrics differentiate honey by country of origin, and examination of changes in NMR spectra as a function of adulteration with sugar syrups^48,49^. Peer-reviewed publication of this industry-led work would add meaningfully to the collective ability to deal with the issues discussed herein.

Meanwhile polarised positions remain unresolved as illustrated in the press article^2^ that prompted this study. There is consensus agreement that multiple approaches are needed to assess honey authenticity, leading inevitably to complex and data-rich certificates of analysis. These are difficult to interpret without further information and an in-depth knowledge of the techniques involved and hence are largely opaque to all but a small defined community of specialists. The summary opinion of the reporting laboratory in each of the CoA examined herein was unequivocally that the samples were noncompliant. However our critical examination of the CoA data reveals a much more nuanced picture from which it is currently difficult to draw a definitive opinion on the authenticity of the samples examined.

Many of the conventional, harmonised physicochemical methods underpinning limits in the EU Directive cannot identify sophisticated adulteration. Of the more advanced techniques, EA-LC-IRMS is well characterised and accepted with known and internationally validated performance characteristics, but there seems little immediate prospect of other reported analytical techniques becoming definitive and accepted.

Without further work and particularly further data disclosure the evidence in the examined CoA for adulteration, including with added sugars, is under-developed. This prompts two questions. (1) When reporting honey authentication data is it acceptable to give an interpretation, particularly an adverse one, without exhibiting all the supporting data? (2) How may a valid overarching opinion on authenticity be derived, from a large partially conflicting dataset? In Part 2 of this work we explore these questions and propose weighted, evidence-based appraisal of results of authenticity analyses by a forensic ‘evaluative reporting’ thought process.

## Supplementary information

Supplementary Figure 1

## Data Availability

All data generated or analysed during this study are included in this published article (and/or its supplementary information files).
