# Supplementary material for: Honey authenticity: the opacity of analytical reports - part 1 defining the problem
Source: NPJ Sci Food. 2022 Feb 8;6:11. doi: 10.1038/s41538-022-00126-6 (PMC8825849; doi:10.1038/s41538-022-00126-6)

**Supplementary Figure 1**

Summary of authentication techniques and the number of samples and/or honey types analysed per technique by each study reviewed.

Inspection of these data confirms little has changed since Anklam 1998 [17] noted a prevalence of low numbers of samples in peer reviewed published studies, Source: Drawn by MW from data in Chin and Sowndhararajan 2020 [35]

(a) Sensory techniques, 6 studies, for one of which no sample numbers were reported

(b) Physicochemical techniques, 12 studies

(c) Chromatographic techniques, sugar profiles, 7 studies

(d) Chromatographic techniques, amino acid profiles, 6 studies

(e) Chromatographic techniques, phenolic and flavonoid compounds, 15 studies

(f) Chromatography-mass spectrometry, studies of volatile compounds (19) and sugar profiles (3)

(g) IRMS, 15 studies for 2 of which no sample numbers reported

(h) Spectroscopic techniques other than NMR

(i) NMR, 14 studies

(j) Molecular techniques, 14 studies for which 6 had no sample numbers reported

(k) Assorted other techniques, 11 studies

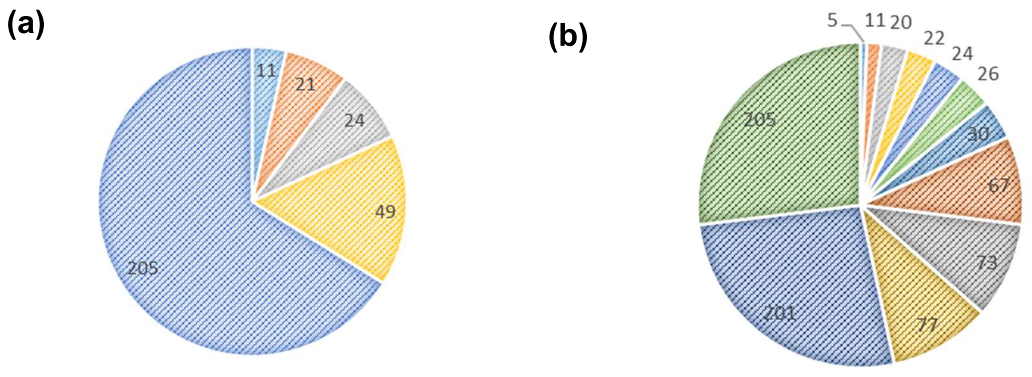

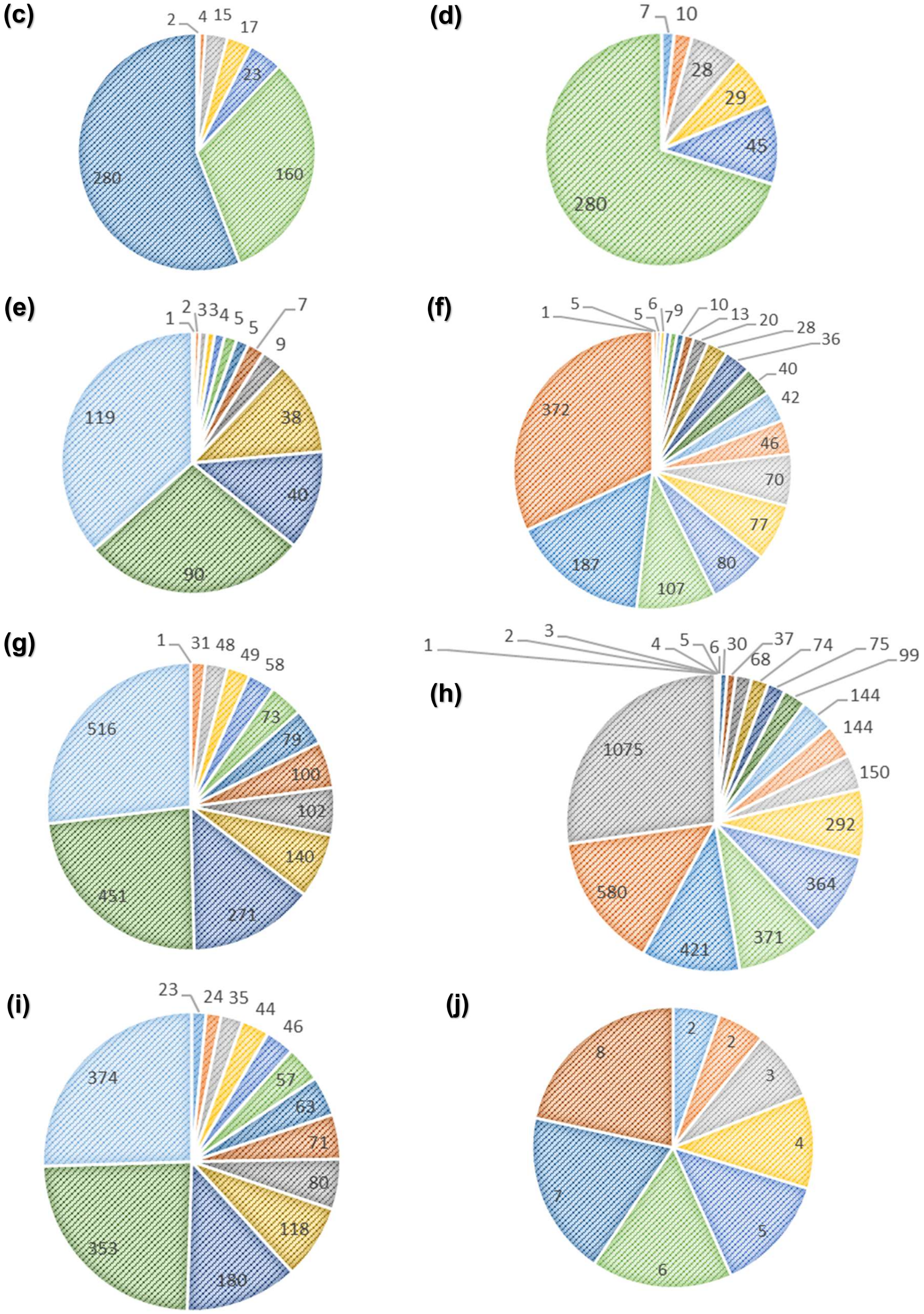

(c)
2
4
15
17
23
160
280
(d)
7
10
28
29
45
280
(e)
1
2
33
4
5
5
7
9
38
40
90
119
(f)
1
5
5
6
7
9
10
13
20
28
36
40
42
46
70
77
80
107
187
372
(g)
1
31
48
49
58
73
79
100
102
140
271
451
516
(h)
1
2
3
4
5
6
30
37
68
74
75
99
144
144
150
292
364
371
421
580
1075
(i)
23
24
35
44
46
57
63
71
80
118
180
353
374
(j)
2
2
3
4
5
6
7
8

(k)

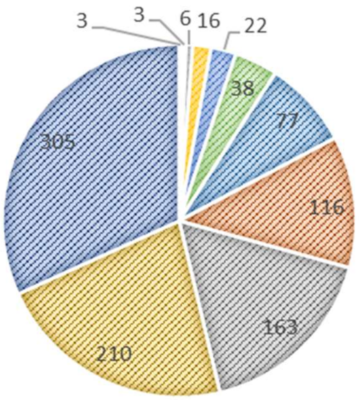

Supplement: Supplementary file 1 — Supplementary Figure 1 [file 41538_2022_126_MOESM1_ESM.pdf]
